# Green Synthesis of Silver Nanoparticles using *Bidens Frondosa *Extract and their Tyrosinase Activity

**Published:** 2017

**Authors:** Qamar Abbas, Muhammad Saleem, Abdul Rehman Phull, Muhammad Rafiq, Mubashir Hassan, Ki-Hwan Lee, Sung-Yum Seo

**Affiliations:** a *Department of Biology, Kongju National University, Gongju, Chungnam 314-701, Republic of Korea.*; b *Department of Chemistry, Kongju National University, Gongju, Chungnam 314-701, Republic of Korea.*

**Keywords:** *Bidens frondosa*, Medicinal Plants, Silver Nanoparticles, Tyrosinase Activity

## Abstract

Herbal nanoparticles gain lot of attention because of their pharmaceutical importance. The present study reports the eco-friendly synthesis, characterization and their tyrosinase activity of silver nanoparticles (AgNPs) using aqueous extract of* Bidens frondosa. *The appearance of brown color indicated the formation of *B. frondosa *AgNPs. The Formation of AgNPs was confirmed by UV–Vis spectroscopy, FTIR, FESEM and EDS analysis. The formation of herbal AgNPs of size ranging 20-70 nm further was assured by energy dispersive X-ray spectroscopy (EDS) and field emission scanning electron microscopy (FESEM). The mushroom tyrosinase inhibitory activity of synthesized AgNPs was evaluated. Nanoparticles were found to have significant higher tyrosinase inhibitory activity compared to control. The IC_50_ values of crude extract, AgNP and Kojic acid were found to be 9, 15, and 2.37 μg/mL, respectively. AgNPs of *B. frondosa *may be considered as potential candidate for the production of medical and cosmetic products.

## Introduction

Herbal medications have been widely used all over the world since ancient times and have been recognized by physicians and patients for their better therapeutic value as they have less side effects as compared with modern medicines. Metal nanoparticles have gained a lot of attention in the recent years due to their unique optical, electrical, and biological properties, which make them central to numerous uses such as in catalysis, bio-sensing, imaging, drug delivery and optical spectroscopy including surface-enhanced Raman scattering (SERS) (1). Among various types of metal nanoparticles such as copper, zinc, and gold, Ag are of more interest due to their chemical properties and biological importance. Plants, which are rich in bioactive compounds, have recently been used for the green synthesis of nanoparticles (2). Synthesis of silver and other metallic nanoparticles from natural sources is being widely explored by using different parts of plants as fruits (3), flowers (4) and leaves (5). Plant mediated synthesized silver nanoparticles are reported to have antimicrobial activity as silver nanoparticles (AgNPs) of *Argimone maxicana* leaves were found to be active against pathogenic fungal and bacterial strains (6). AgNPs exhibit outstanding physical, chemical and biological properties. AgNPs have potential in treating a variety of diseases, including retinal neovascularization, and immunodeficiency syndrome (7). 

Tyrosinase plays critical role in melanin biosynthesis, which is responsible for pigmentation in mammals. Synthesis of tyrosinase inhibitors through green chemistry approach reported to have lower side effects comparative to synthetic inhibitors (8). There is tremendous interest to cosmetic industry for development of cost effective, natural and prospective antityrosinase for bleaching-off extremely pigmented lesions. Hence, the development of agents for bleaching-off hyper-pigmentation, which is with little or no side effects, of natural origin, cost-effective, as well as being anti-melanoma and anti-microbial, is of tremendous interest to the cosmetic industry (9).

*Bidens frondosa *L originally occurs in North America and grows at the shore of lakes and rivers, in nutrient-rich mud, or in sand soils (10). Fresh stems and leaves of *B. frondosa *are used as human food commonly known as devils beggarticks. Species of *Bidens* genus possess various pharmacological activities like antidiabetic, anti-inflammatory, anticancer, anti-viral and other activities (11).

Herein we report eco-friendly synthesis, characterization and their tyrosinase inhibition of AgNPs using aqueous extract of *B. frondosa*.

## Experimental

*Materials and Methods*

*Selection of plant material*

The *B. frondosa *plant was obtained from “Korean Collection of Herbal Extracts”, a biotech company in Korea. 

*Preparation of the plant extracts*

The whole plant was washed thrice with distilled water to remove the associated contamination. About 40 g of finely cut plant was placed in 500 mL of sterile distilled water and then the mixture was boiled. The boiled extract was filtered with Whatmann filter (110 mm). Filtrated aqueous extract was used for synthesis of silver nanoparticles.

*Synthesis of silver nanoparticles*

Silver nitrate was purchased from Sigma Aldrich (cat. 7761-88-8). Aqueous extract of 50 mL was added in flask and 1 mM aqueous solution of AgNO_3_ was added drop wise until the conversion of solution color from yellow to brown color. Conversion of color indicates the formation of silver nanoparticles.

*Characterization of silver nanoparticles*

The synthesized AgNPs were examined by UV-vis spectroscopic (Shimadzu, Kyoto, Japan) analysis. The shape and size of the synthesized AgNPs were measured using a field emission scanning electron microscope (JEOL JSM 6490 USA). Energy Dispersive X-ray spectrometric analysis (EDS) (MIRA3 LMH, TESCAN Czech, Republic) was performed to find out the elements and their relative weight percent in the synthesized AgNPs. 

*Mushroom tyrosinase assay*

The mushroom tyrosinase (EC 1.14.18.1) (Sigma Chemical Co.) was used for *in vitro* bioassay by repeated procedure with some modifications (12). Briefly, 140 μL of phosphate buffer (20 mM, pH 6.8), 20 μL of mushroom tyrosinase (30 U/mL) and 20 μL of the inhibitor solution were placed in the wells of a 96-well micro plate. After pre-incubation for 10 min at room temperature, 20 μL of L-DOPA (3,4-dihydroxyphenylalanine, 0.85 mM) was added and the plate was further incubated at 25 °C for 20 min. Subsequently the absorbance of dopachrome was measured at 492 nm using a micro plate reader (OPTI _Max_, Tunable). Kojic acid was used as a reference inhibitor and for negative tyrosinase inhibitor phosphate buffer was used instead of the inhibitor solution. The extent of inhibition by the test compounds was expressed as the percentage of concentration necessary to achieve 50% inhibition (IC_50_). Each concentration was analyzed in three independent experiments run in triplicate. The IC_50_ values were determined by the data analysis and graphing software Origin 8.6, 64-bit.

## Results and Discussion

*Photophysical properties*

**Figure 1:**
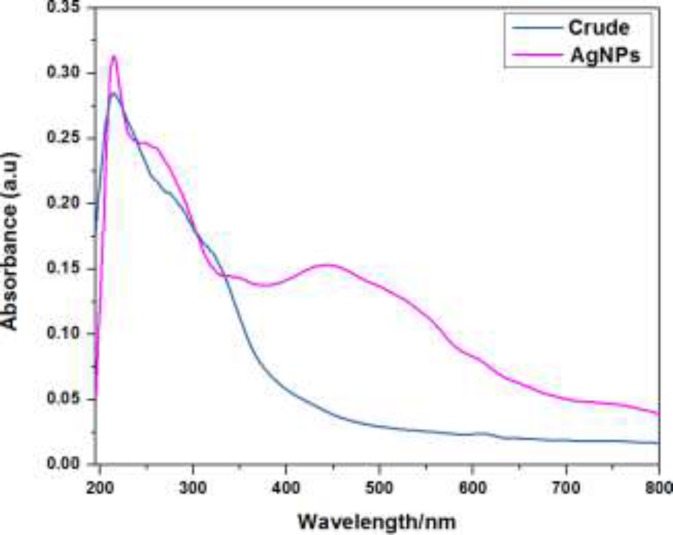
The UV-visible absorption spectra of crude plant extract and AgNPs

The formation and reduction of silver nanoparticles (AgNPs) by plant extract were monitored using UV-visible spectroscopic analysis. During the addition of aqueous AgNO_3_ solution into plant extract, the instantaneous change in color of the solutions from light yellow to dark brown suggested the formation of silver nanoparticles. The plant extract (1 mg/mL) exhibited an absorption signal at 214 nm while there was no absorption signal beyond this in the entire range from 400-750 nm. Interestingly, an intense absorption signal at 443 nm with the considerable molar absorptivity was observed on incubation of plant extract with the aqueous solution of silver nitrate (1 × 10^-5 ^mol L^-1^). This red shifted signal appearance as well as colorimetric change in the plant extract on silver nitrate addition provided the evidence for the formation of AgNPs triggered by the reduction with the plant extract (Figure 1).

**Table 1:** FTIR spectral bands of crude of B. frindosa (CE) and AgNPs

Observed spectral Bands (cm^-1^)		Suggested groups	Biomolecules
CE	AgNPs		
**3414 **	**3361 **	**-OH,N-H Stretch **	**Alcohols, phenols, proteins **
**2924 **	**2924 **	**>CH2, -CH3 **	**Alkanes **
**2848 **	**- **	**-OCH3 C-H stretch **	**Alkanes, aldehydes **
**1604 **	**1635 **	**N-H bend **	**Amines, protein, terpenoids **
**1458 **	**- **	**>CH2,-CH3 C-H bend **	**Alkanes **
**1392 **	**1292 **	**-OH, N-O stretch **	**Phenolics, Nitrocompounds **
**1261 **	**1257 **	**-OH **	**Amides **
**1122 **	**- **	**Alkyl,C-N stretch **	**Aliphatic amines **
**1069 **	**1072 **	**C-Br, **	**Carboxylic acids, esters, ethers Aromatic **
**867 **	**890 **	**-CH **	**Aromatic hydrocarbon **

CE= crude extract of *B. frindosa*

**Figure 2:**
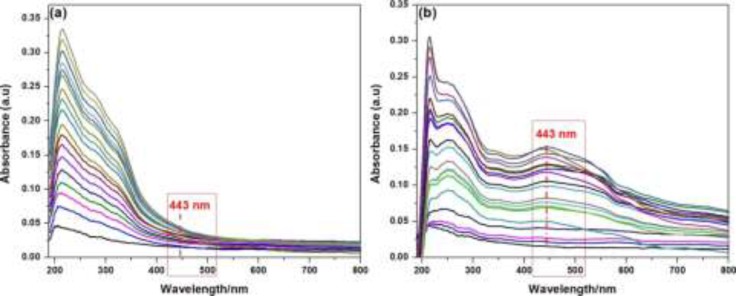
The UV–visible absorption spectra of; (a) crude plant extract with variable concentrations ranging from 1 × 10^-5^ gmol^-1^ to 1 × 10^-3^ gmol^-1^ at ambient temperature without addition of silver nitrate solution (1 × 10^-5^ mol L^-1^, aqueous solution); (b) after addition of 1 × 10^-5^ mol L^-1^ aqueous solution of silver nitrate

**Figure 3:**
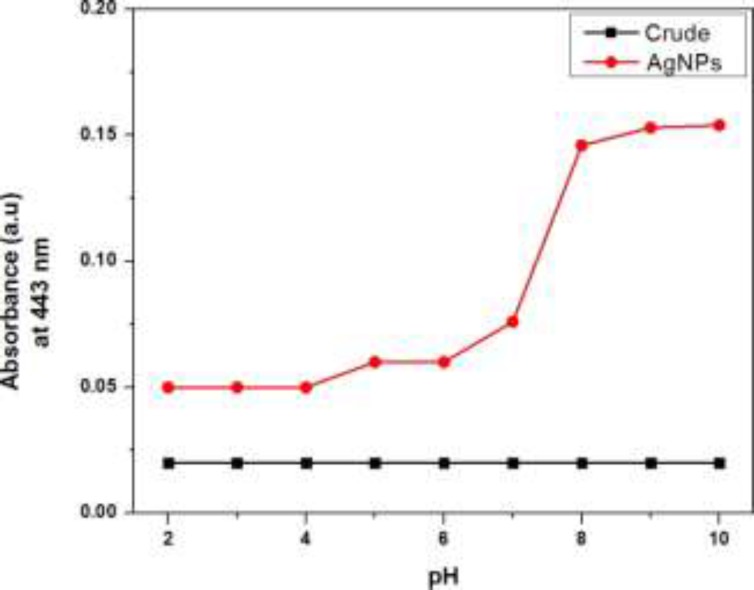
Effect of pH on the absorption signal intensity at 443 nm

**Figure 4:**
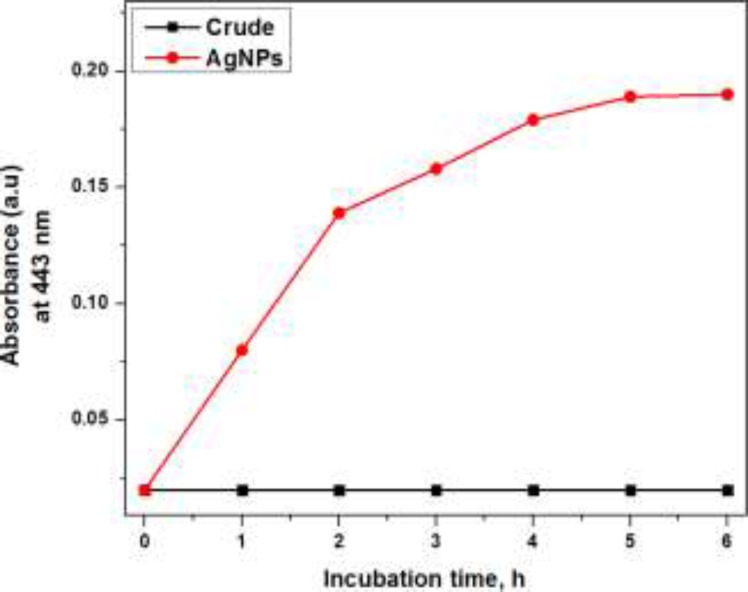
Effect of incubation time on the synthesis of AgNPs

**Figure 5:**
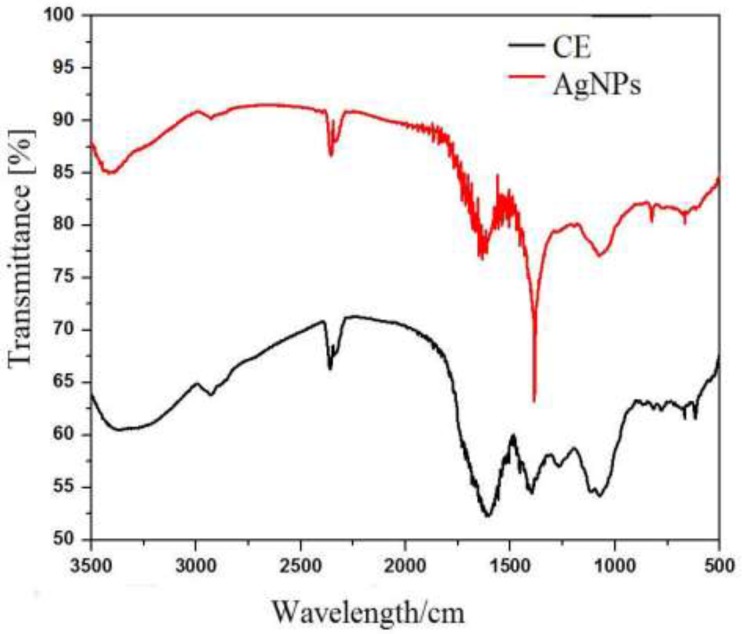
FTIR Spectra of Crude and AgNPs at 500 cm^-1 ^to 3500 cm^-1^

**Figure 6:**
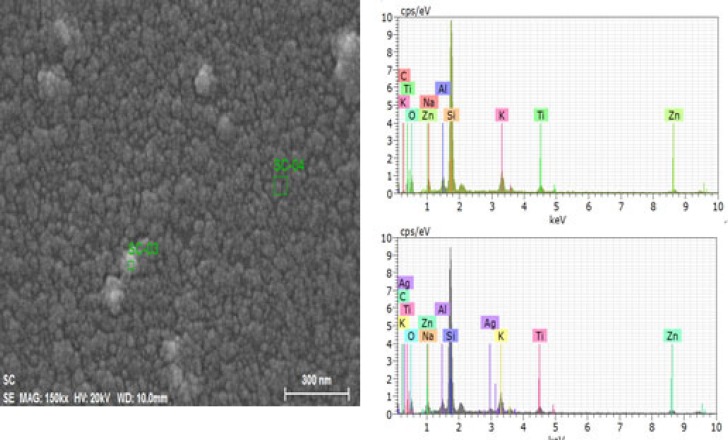
Energy-dispersive X-ray spectrometric (EDS) figures nanoparticles (spectrum: SC-03) Less aggregated area (spectrum: SC-04

**Figure 7:**
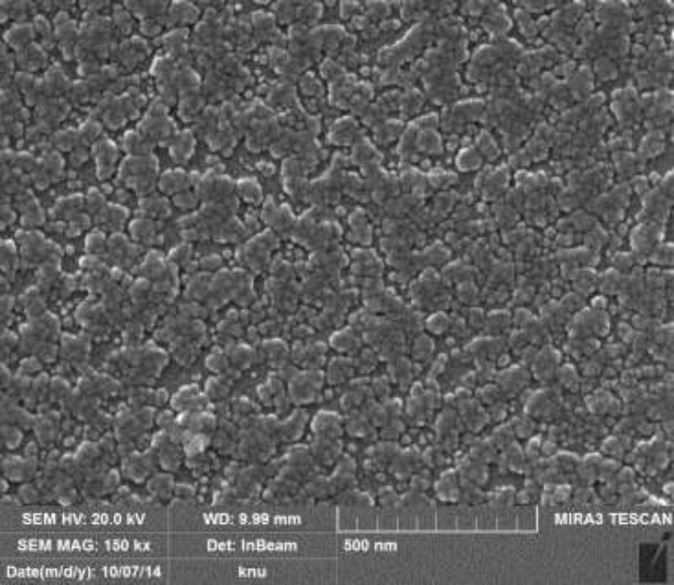
FESEM micrograph of AgNPs of *Biden frondosa*

**Figure 8:**
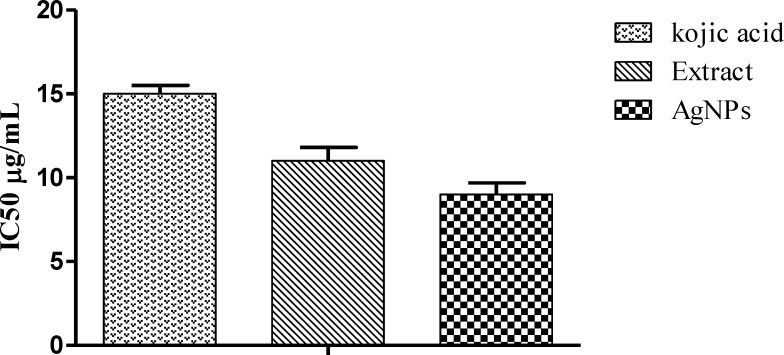
Tyrosinase Inhibition by aqueous crude extract and AgNPs while kojiac acid used as Reference

*Effect of concentration*

In order to monitor the formation and stability of silver nanoparticles, the UV-visible absorption spectra were recorded in the absence and presence of fixed concentration of AgNO_3_^2-^ (1 × 10^-5 ^mol L^-1^, aqueous solution) with variable concentration of crude plant extract ranging from 1 × 10^-5 ^g L^-1^ to 1 × 10^-3^ g L^-1^ at ambient temperature. The color of the solutions changed from light yellow to pale yellow on increasing crude extract concentration in the absence of silver nitrate solution. But in the presence of silver nitrate, with the increase of extract concentration the crude extract color change from yellowish to brown and further to deep brown due to excitation of surface Plasmon vibration, indicating formation in the silver nanoparticles. Absorbance peak at 443 nm may be attributed to the surface plasmon resonance of AgNPs and earlier report suggest that noble metal particles as silver showed the characteristic peak around 430 nm (13). Meanwhile, the extract concentrations showed the effect on the extract absorption signals intensity at 214 nm as shown in Figure 2. There was ratiometric increment in the extract absorption signal intensity at 214 nm. However, the same reaction solution in the presence of AgNO_3_^2-^ (1 × 10^-5^mol L^-1^, aqueous solution) exhibited a new absorption signal centered at 443 nm. This red shifted absorption signal and colorimetric change in the reaction solution provided evidence for the successful formation of silver nanoparticles by the bio-reduction of silver ion.

*Effect of pH*

To get better sagaciousness for the successful synthesis and better yield of AgNPs, the proper pH condition was optimized by recording the UV-visible absorption spectra in the buffer solution with the variable pH range from 2 to 10 as shown in Figure 3. The intensity of signal at 443 nm characteristic for the silver nanoparticles was greatly influenced by pH values. The maximum intensity absorption signal at 443 nm was obtained in the entire alkaline conditions while in case of acidic pH, there was dramatic downfall in the signal intensity at 443 nm. AgNPs formation was preferred in the alkaline conditions as the constituents of extract in the alkaline pH conditions possess maximum number of negatively charged functional groups capable of efficient binding and reducing the silver ion (14). 

*Effect of incubation time*

The maximum absorbance at 443 nm in case of UV-visible absorption spectra appeared on increasing with the incubation time of crude plant extract with aqueous silver nitrate solution and became constant on 5 h of incubation as shown in Figure 4. This finding suggests that the AgNPs formation started immediately on incubating the crude plant extract with silver nitrate solution and maximum synthesis of AgNPs occurred on 5 h of incubation at ambient temperature while afterward the reaction mixture become saturated as there was no considerable increment in the absorption signal intensity at 443 nm. Appropriate incubation time span is required for reduction of silver ion (15, 16).

*Fourier Transform Infrared (FTIR) Analysis*

Fourier transforms infrared (FTIR) measurement of AgNP synthesized by using *b. frondosa* extract. It was carried to detect the synthesis of nanoparticles. The IR band apparent at 3409 cm^-1^ in extract is characteristic of the O–H and it was shifted to 3361 cm^-1^ in AgNP. While other prominent shift in the wave numbers corresponding to amide (1604 to1635 cm^-1^) suggested that involvement of amino (–NH_2_) or COO^- ^(carboxylate) in crude extract making surface AgNPs stable. The medium IR band observed in extract at 1604 cm^-1^ corresponds to amide arising due to carbonyl stretch was shifted to 1635 cm^-1 ^in AgNPs. Other miner shifts in the IR bands from 2369 to 2355 cm^-1^ and 2332 to 2328 cm^-1^ suggest the reaction of silver ions with extract (Table1). 

In addition the observed bands indicate the presence of terpenoids which are important in reduction of metal ions by the oxidizing aldehyde groups to carboxylic acids (17). Bioactive compounds as phenolics and proteins different plants are reported to have role in formation of nanoparticles. Involvements of free amino acids of proteins in AgNPs are reported (18). These observations suggest the role of biomolecule in the synthesis of nanoparticles. It is therefore the possibility of involvement of proteins and aromatic compounds in the stabilization and binding in AgNPs (19).

*Energy-dispersive X-ray spectrometric (EDS) analysis*

Nanoparticles and crude sample were investigated for elemental analysis and presence of silver using energy dispersive X-ray spectrometric (EDS) method. Elements shown in EDS spectrum are the constituents of the extract used for the synthesis of nanoparticles. While distinct signals in EDS spectrum in silver region confirms the synthesis of the nanoparticles (Figure 5)

*Field emission scanning electron microscopy (FESEM)*

The Figure 6 showed the result of FESEM. Most of the Ag-NPs were spherical in shape and the sizes of AgNPs were ranges from 20-70 nm but most of them were 30 nm. 

*Mushroomtyrosinase activity*

Mushrom tyrosinase enzyme was used in preliminary screening. Chelation of copper ions in mushroom tyrosinase determines the inhibitory potential of the sample. AgNPs showed enhanced inhibitory activity comparative to extract and standard. The IC_50_ values were found to be 9, 11, and 2.37 μg/mL for AgNPs, plant extract and kojic acid, respectively (Figure 7). The tyrosinase inhibitory activity might be attributed towards the bioactive compounds like phenolic and flavonoids involved in nanoparticle synthesis (20).

## Conclusions

Synthesis of nanoparticles by using plant is an eco-friendly, fast and inexpensive process. In this study the AgNPs were synthesized using aqueous extract of *B. frondosa*. The synthesized AgNPs were characterized by UV-vis spectroscopy, FTIR, EDS and FESEM. The AgNPs were proved to be potent inhibitors of tyrosinase. The green synthesis of AgNPs could be immensely useful in cosmetic and pharmaceutical industries. 
